# Correction: Depression and physical multimorbidity: A cohort study of physical health condition accrual in UK Biobank

**DOI:** 10.1371/journal.pmed.1004740

**Published:** 2025-09-11

**Authors:** Kelly J. Fleetwood, Bruce Guthrie, Caroline A. Jackson, Paul A. T. Kelly, Stewart W. Mercer, Daniel R. Morales, John D. Norrie, Daniel J. Smith, Cathie Sudlow, Regina Prigge

In the Funding statement, the grant number from the funder Medical Research Council (https://www.ukri.org/councils/mrc/)/National Institute for Health Research (https://www.nihr.ac.uk/) is listed incorrectly. The correct grant number is: MR/S028013/1.
