# Supplementary material for: Depression and physical multimorbidity: A cohort study of physical health condition accrual in UK Biobank
Source: PLoS Med. 2025 Feb 13;22(2):e1004532. doi: 10.1371/journal.pmed.1004532 (PMC11825000; doi:10.1371/journal.pmed.1004532)
Supplement: S1 Text — (DOCX) [file pmed.1004532.s002.docx]

**Supporting Information: S1 Text**

**Depression and physical multimorbidity: a cohort study of physical health condition accrual in UK Biobank**

Kelly J Fleetwood^1^*^¶^, Bruce Guthrie^2¶^, Caroline A Jackson^1^, Paul AT Kelly^3^, Stewart W Mercer^1^, Daniel R Morales^4^, John D Norrie^1,#a^, Daniel J Smith^5^, Cathie Sudlow^1,6^, Regina Prigge^1^

^1^ Usher Institute, University of Edinburgh, Edinburgh, UK

^2^ Advanced Care Research Centre, Usher Institute, University of Edinburgh, Edinburgh, UK

^3^ Public member of study advisory board, Edinburgh, UK

^4^ Division of Population Health and Genomics, University of Dundee, Dundee, UK

^5^ Centre for Clinical Brain Sciences, University of Edinburgh, Edinburgh, UK

^6^ Health Data Research UK, London, UK

^#a^ Current address: Queen’s University, Belfast, UK

* Corresponding author

Email: [kelly.fleetwood@ed.ac.uk](mailto:kelly.fleetwood@ed.ac.uk)

^¶^ These authors contributed equally to this work

Contents

[Table A: Availability of electronic heath records 3](#_Toc184052140)

[Table B: Long-term physical health conditions included in our counts of conditions at baseline and during follow-up 4](#_Toc184052141)

[Table C: Long-term mental health conditions included in our count of conditions at baseline 7](#_Toc184052142)

[Table D: Additional information about covariate definition 8](#_Toc184052143)

[Figure A: Proportional Venn diagram for history of depression at baseline by data source 9](#_Toc184052144)

[Table E: Number and percentage of people with a history of each long-term physical health condition at UKB baseline assessment and diagnosed during follow-up, stratified by history of depression at baseline (N = 172,556) 9](#_Toc184052145)

[Table F: Number and percentage of people with a history of each long-term physical health condition at baseline, stratified by history of depression at baseline (N = 172,556). Conditions present from birth 12](#_Toc184052146)

[Table G: Number and percentage of people with a history of each long-term mental health condition at baseline, stratified by history of depression at baseline (N = 172,556). 13](#_Toc184052147)

[Table H: Rate ratios for the association of history of depression at baseline, sociodemographic, social, lifestyle and clinical factors with physical health condition accrual during follow-up. Complete cases analysis (N = 132,394). 14](#_Toc184052148)

[References 15](#_Toc184052149)

# Table A: Availability of electronic heath records

| **Data source** | **Country** | **Earliest record** | **Complete to** |
| --- | --- | --- | --- |
| Primary care | England | December 1937***** | 31 May 2016 |
|  | Scotland | August 1937***** | 31 March 2017 |
|  | Wales | May 1940***** | 31 August 2017 |
| Hospital admission | England | April 1997 | 31 October 2022 |
|  | Scotland | January 1981 | 31 August 2022 |
|  | Wales | January 1998† | 31 May 2022 |
| Cancer registry | England | 1971 | 31 December 2020 |
|  | Scotland | 1957 | 30 November 2021 |
|  | Wales | 1971 | 31 December 2016 |
| Death records | England | 2006 | 30 November 2022 |
|  | Scotland | 2006 | 30 November 2022 |
|  | Wales | 2006 | 30 November 2022 |

* Excluding records prior to each participant’s date of birth

†At time of publication, the UK Biobank website (<https://biobank.ndph.ox.ac.uk/showcase/exinfo.cgi?src=Data_providers_and_dates>) gives the start date for Wales as 1991, but data records aren’t consistently available until 1 Jan 1998

# Table B: Long-term physical health conditions included in our counts of conditions at baseline and during follow-up

| **Condition** | **Condition includes** |
| --- | --- |
| Benign neoplasm of brain and other CNS |  |
| Haematological malignancies | Hodgkin lymphoma |
|  | Leukaemia |
|  | Monoclonal gammopathy of undetermined significance (MGUS) |
|  | Multiple myeloma and malignant plasma cell neoplasms |
|  | Myelodysplastic syndromes |
|  | Non-Hodgkin lymphoma |
|  | Polycythaemia vera |
|  | Haematological malignancy - other |
| Non-melanoma skin malignancies |  |
| Solid organ malignancies | Primary malignancy - biliary tract |
|  | Primary malignancy - bladder |
|  | Primary malignancy - bone and articular cartilage |
|  | Primary malignancy - brain, other central nervous system and intracranial |
|  | Primary malignancy - breast |
|  | Primary malignancy - cervical |
|  | Primary malignancy - colorectal and anus |
|  | Primary malignancy - kidney and ureter |
|  | Primary malignancy - liver |
|  | Primary malignancy - lung and trachea |
|  | Primary malignancy - malignant melanoma |
|  | Primary malignancy - mesothelioma |
|  | Primary malignancy - multiple independent sites |
|  | Primary malignancy - oesophageal |
|  | Primary malignancy - oro-pharyngeal |
|  | Primary malignancy - ovarian |
|  | Primary malignancy - pancreatic |
|  | Primary malignancy - prostate |
|  | Primary malignancy - stomach |
|  | Primary malignancy - testicular |
|  | Primary malignancy - thyroid |
|  | Primary malignancy - uterine |
|  | Primary malignancy - other organs |
|  | Secondary malignancy - adrenal gland |
|  | Secondary malignancy - bone |
|  | Secondary malignancy - bowel |
|  | Secondary malignancy - brain, other central nervous system and intracranial |
|  | Secondary malignancy - liver and intrahepatic bile duct |
|  | Secondary malignancy - lung |
|  | Secondary malignancy - lymph nodes |
|  | Secondary malignancy - pleura |
|  | Secondary malignancy - retroperitoneum and peritoneum |
|  | Secondary malignancy - other organs |
| Cardiomyopathy | Dilated cardiomyopathy |
|  | Hypertrophic cardiomyopathy |
|  | Other cardiomyopathy |
| Conduction disorders and other arrhythmias | Atrioventricular block, complete |
|  | Sick sinus syndrome |
|  | Supraventricular tachycardia |
| Coronary heart disease | Coronary heart disease not otherwise specified |
|  | Myocardial infarction |
|  | Stable angina |
|  | Unstable angina |
| Heart valve disorders | Multiple valve disorder |
|  | Nonrheumatic aortic valve disorders |
|  | Nonrheumatic mitral valve disorders |
|  | Rheumatic valve disorder |
| Stroke | Intracerebral haemorrhage |
|  | Ischaemic stroke |
|  | Stroke not otherwise specified |
| Atrial fibrillation |  |
| Heart failure |  |
| Hypertension |  |
| Peripheral arterial disease |  |
| Primary pulmonary hypertension |  |
| Transient ischaemic attack |  |
| Chronic liver disease | Alcoholic liver disease |
|  | Autoimmune liver disease |
|  | Hepatic failure |
|  | Liver fibrosis, sclerosis and cirrhosis |
|  | Portal hypertension |
|  | Chronic viral hepatitis |
| Gastro-oesophageal reflux, gastritis and similar [abbreviated to gastro-oesophageal reflux disease (and similar) in the main text] | Barrett's oesophagus |
|  | Gastritis and duodenitis |
|  | Gastro-oesophageal reflux disease |
|  | Oesophagitis and oesophageal ulcer |
| Inflammatory bowel disease | Crohn's disease |
|  | Ulcerative colitis |
| Coeliac disease |  |
| Diverticular disease of intestine (acute and chronic) |  |
| Fatty liver |  |
| Irritable bowel syndrome |  |
| Peptic ulcer disease |  |
| Hearing loss |  |
| Meniere disease |  |
| Addison’s disease |  |
| Cystic fibrosis* |  |
| Hypo or hyperthyroidism |  |
| Type 1 diabetes |  |
| Type 2 diabetes |  |
| Diabetes not otherwise specified |  |
| Glaucoma |  |
| Macular degeneration |  |
| Visual impairment and blindness |  |
| Chronic renal disease | Chronic kidney disease |
|  | End stage renal disease |
|  | Glomerulonephritis |
|  | Tubulo-interstitial nephritis |
| Erectile dysfunction |  |
| Hyperplasia of prostate |  |
| Non-acute cystitis |  |
| Urinary incontinence |  |
| Allergic and chronic rhinitis |  |
| Asbestosis |  |
| Asthma |  |
| Bronchiectasis |  |
| Chronic obstructive pulmonary disease |  |
| Sleep apnoea |  |
| Iron and vitamin deficiency anaemia | Folate deficiency anaemia |
|  | Iron deficiency anaemia |
|  | Vitamin B12 deficiency anaemia |
| Immunodeficiencies |  |
| Sarcoidosis |  |
| Sickle-cell anaemia* |  |
| Thalassaemia* |  |
| HIV |  |
| Tuberculosis |  |
| Inflammatory arthritis and other inflammatory conditions | Ankylosing spondylitis |
|  | Juvenile arthritis* |
|  | Lupus erythematosus (local and systemic) |
|  | Polymyalgia rheumatica |
|  | Postinfective and reactive arthropathies |
|  | Psoriatic arthropathy |
|  | Rheumatoid arthritis |
|  | Systemic sclerosis |
| Osteoporosis and vertebral crush fractures | Collapsed vertebra |
|  | Osteoporosis |
| Gout |  |
| Osteoarthritis (excl spine) |  |
| Spinal stenosis |  |
| Peripheral or autonomic neuropathy | Diabetic neurological complications |
|  | Disorders of autonomic nervous system |
|  | Peripheral neuropathies (excluding cranial nerve and carpal tunnel syndromes) |
| Cerebral palsy* |  |
| Epilepsy |  |
| Migraine |  |
| Motor neurone disease |  |
| Multiple sclerosis |  |
| Myasthenia gravis |  |
| Parkinson's disease |  |
| Post-viral fatigue syndrome, neurasthenia and fibromyalgia |  |
| Down's syndrome* |  |
| Psoriasis |  |
| Dementia |  |

CNS, central nervous system; HIV, human immunodeficiency virus.

* Condition present from birth

# Table C: Long-term mental health conditions included in our count of conditions at baseline

| **Condition** |
| --- |
| Alcohol problems |
| Anorexia and bulimia nervosa |
| Anxiety disorders |
| Autism and Asperger's syndrome* |
| Bipolar affective disorder and mania |
| Intellectual disability* |
| Obsessive-compulsive disorder |
| Other psychoactive substance misuse |
| Post-traumatic stress disorder |
| Schizophrenia, schizotypal and delusional disorders |

* Condition present from birth

# Table D: Additional information about covariate definition

| Variable | Definition |
| --- | --- |
| Stressful life events | We counted the number of stressful life events in the two years prior to the baseline assessment including: serious illness/injury/assault of a close relative, death of a close relative, death of a spouse/partner, marital separation/divorce and financial difficulties, but excluding serious illness/injury/assault of the participant, since we used the count of long-term health conditions to measure serious illness. |
| Loneliness | We measured loneliness based on whether the participant answered yes or no to the question ‘do you often feel lonely?’ |
| Chronic multi-site pain | We defined chronic multi-site pain as having pain in at least two body sites or all over the body for at least 3 months. |
| Sleep | We measured sleep based on the question ‘do you have trouble falling asleep at night or do you wake up in the middle of the night?’ with responses of never/rarely, sometimes or usually. |
| Smoking status | We used the UK Biobank smoking status summary field to identify current, previous and never smokers. |
| Frailty | We included four covariates related to frailty:^1^ weight loss, slow walking speed, weak grip strength and low physical activity. Whilst exhaustion is included in some frailty measures, we didn’t include it as a covariate because it is a symptom of depression. |

# Figure A: Proportional Venn diagram for history of depression at baseline by data source

| 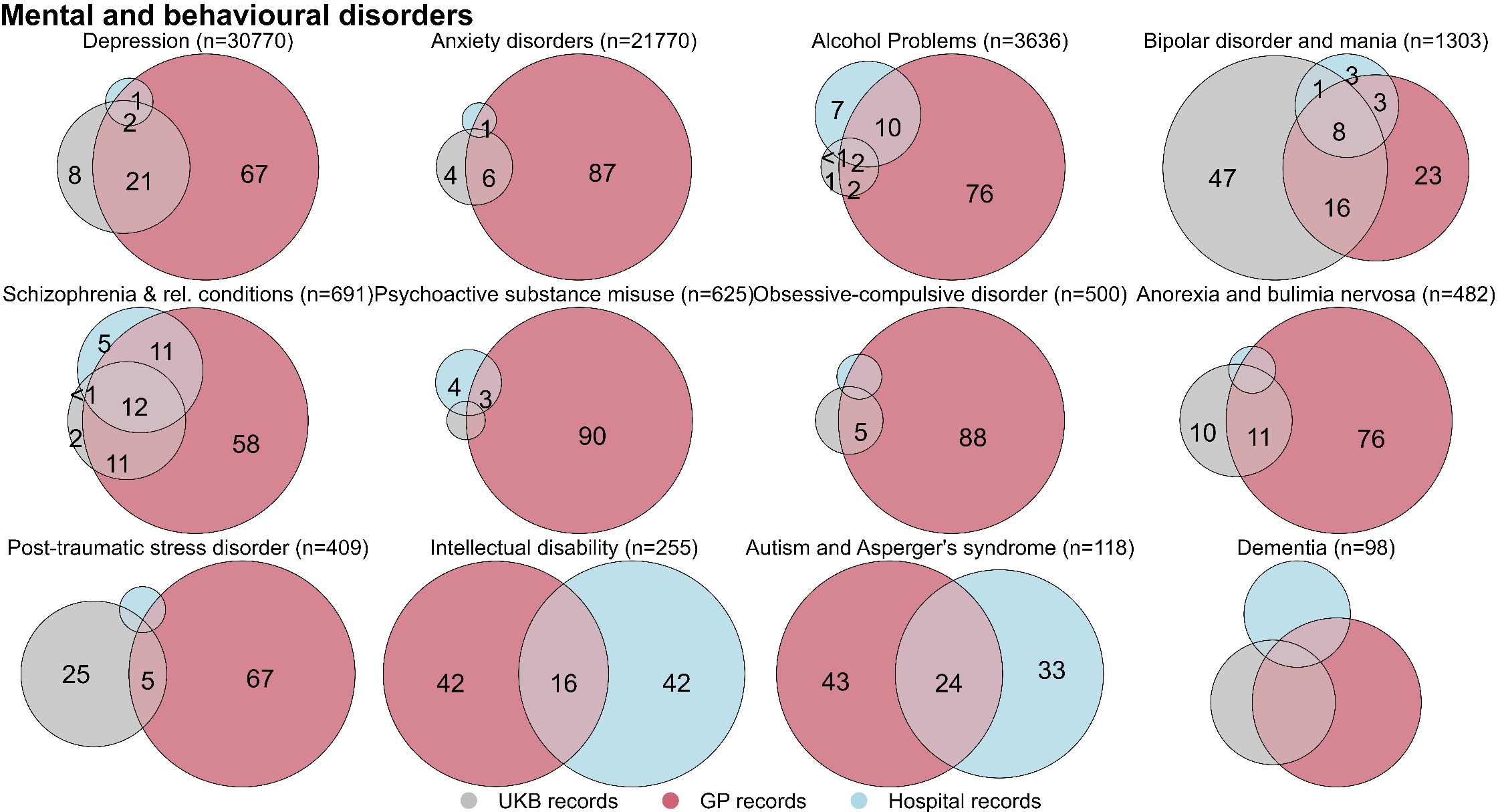 | 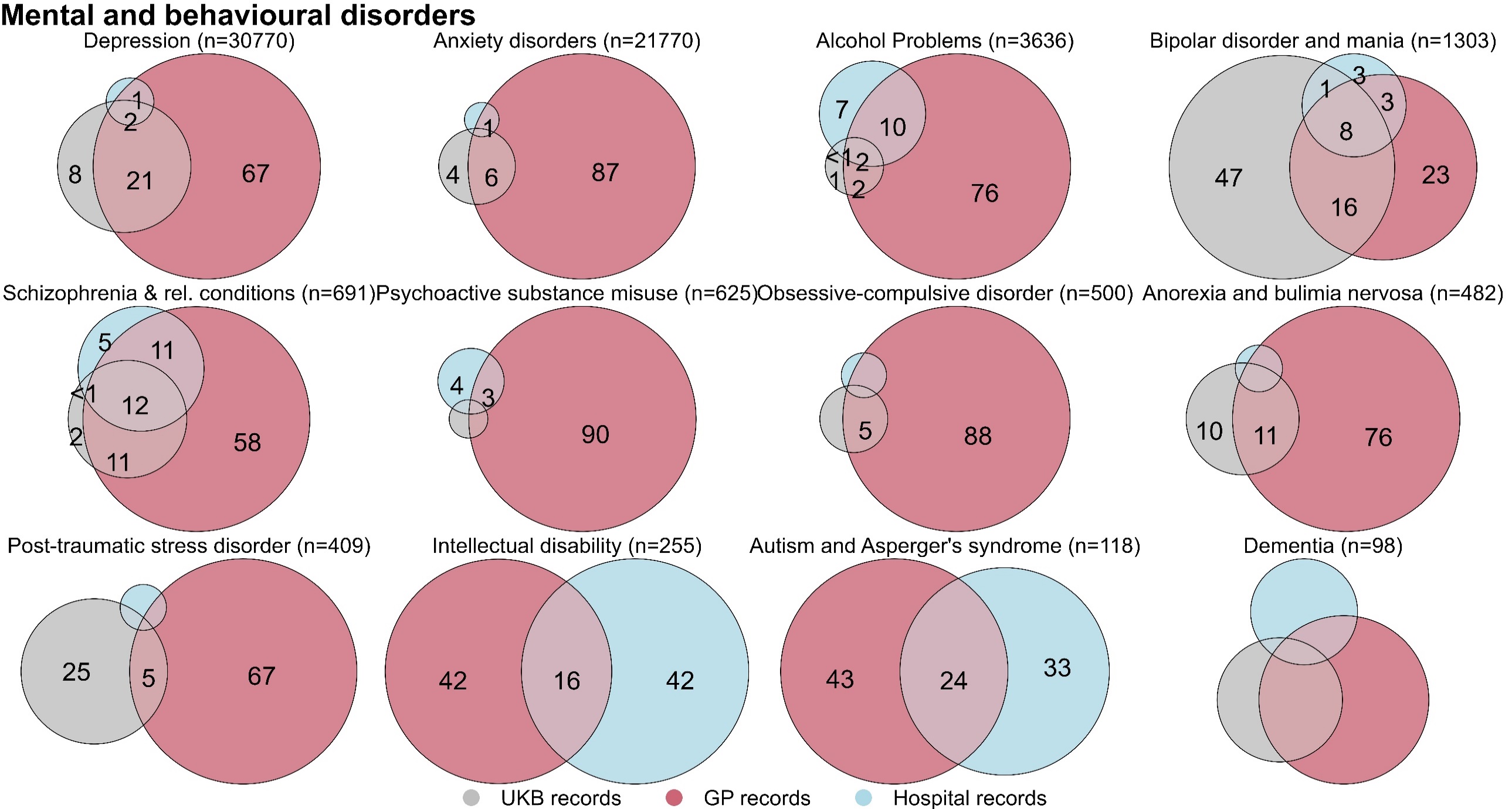 |
| --- | --- |

# Table E: Number and percentage of people with a history of each long-term physical health condition at UKB baseline assessment and diagnosed during follow-up, stratified by history of depression at baseline (N = 172,556)

|  | History of physical LTC at UKB baseline assessment  [n (%)] | | Physical LTC during follow-up  [n (% of people without a history of the condition at baseline)] | |
| --- | --- | --- | --- | --- |
|  | History of depression at baseline | | | |
| Condition* | Depression (N = 30,770) | No depression (N = 141,786) | Depression | No depression |
| Osteoarthritis (excluding spine) | 8,301 (27.0%) | 27,116 (19.1%) | 3,522 (15.7%) | 14,296 (12.5%) |
| Hypertension | 10,277 (33.4%) | 41,980 (29.6%) | 2,643 (12.9%) | 11,957 (12.0%) |
| Gastro-oesophageal reflux, gastritis and similar | 7,041 (22.9%) | 19,499 (13.8%) | 3,268 (13.8%) | 11,782 (9.6%) |
| Erectile dysfunction† | 1,606 (15.8%) | 6,314 (9.2%) | 1,014 (11.8%) | 5,486 (8.8%) |
| Hyperplasia of prostate† | 1,100 (10.8%) | 5,313 (7.8%) | 824 (9.1%) | 4,643 (7.4%) |
| Solid organ malignancies | 2,421 (7.9%) | 9,535 (6.7%) | 1,729 (6.1%) | 8,249 (6.2%) |
| Diverticular disease of intestine (acute and chronic) | 1,662 (5.4%) | 5,065 (3.6%) | 1,976 (6.8%) | 7,147 (5.2%) |
| Chronic renal disease | 781 (2.5%) | 2,889 (2.0%) | 1,882 (6.3%) | 6,796 (4.9%) |
| Coronary heart disease | 2,680 (8.7%) | 9,486 (6.7%) | 1,398 (5.0%) | 5,824 (4.4%) |
| Type 2 diabetes | 1,766 (5.7%) | 6,180 (4.4%) | 1,293 (4.5%) | 4,875 (3.6%) |
| Allergic and chronic rhinitis | 9,963 (32.4%) | 37,867 (26.7%) | 914 (4.4%) | 3,431 (3.3%) |
| Non-melanoma skin malignancies | 829 (2.7%) | 4,518 (3.2%) | 883 (2.9%) | 4,784 (3.5%) |
| Osteoporosis and vertebral crush fractures | 1,169 (3.8%) | 3,823 (2.7%) | 1,211 (4.1%) | 4,177 (3.0%) |
| Iron and vitamin deficiency anaemia | 1,798 (5.8%) | 5,402 (3.8%) | 1,292 (4.5%) | 3,772 (2.8%) |
| Atrial fibrillation | 552 (1.8%) | 2,668 (1.9%) | 943 (3.1%) | 4,157 (3.0%) |
| COPD | 989 (3.2%) | 2,228 (1.6%) | 1,145 (3.8%) | 3,167 (2.3%) |
| Hypo or hyperthyroidism | 3,016 (9.8%) | 8,995 (6.3%) | 789 (2.8%) | 2,796 (2.1%) |
| Asthma | 5,413 (17.6%) | 18,041 (12.7%) | 777 (3.1%) | 2,515 (2.0%) |
| Urinary Incontinence | 2,518 (8.2%) | 4,815 (3.4%) | 1,079 (3.8%) | 2,459 (1.8%) |
| Gout | 822 (2.7%) | 4,599 (3.2%) | 553 (1.8%) | 2,859 (2.1%) |
| Irritable bowel syndrome | 4,444 (14.4%) | 8,926 (6.3%) | 778 (3.0%) | 2,029 (1.5%) |
| Inflammatory arthritis and other inflammatory conditions | 1,337 (4.3%) | 4,351 (3.1%) | 635 (2.2%) | 2,184 (1.6%) |
| Migraine | 3,969 (12.9%) | 10,000 (7.1%) | 660 (2.5%) | 1,830 (1.4%) |
| Heart failure | 325 (1.1%) | 1,130 (0.8%) | 562 (1.8%) | 2,093 (1.5%) |
| Stroke | 865 (2.8%) | 2,427 (1.7%) | 528 (1.8%) | 2,011 (1.4%) |
| Peripheral or autonomic neuropathy | 631 (2.1%) | 1,663 (1.2%) | 590 (2.0%) | 1,827 (1.3%) |
| Glaucoma | 622 (2.0%) | 2,672 (1.9%) | 420 (1.4%) | 1,974 (1.4%) |
| Heart valve disorders | 549 (1.8%) | 2,133 (1.5%) | 442 (1.5%) | 1,897 (1.4%) |
| Transient ischaemic attack | 482 (1.6%) | 1,582 (1.1%) | 450 (1.5%) | 1,677 (1.2%) |
| Fatty liver | 237 (0.8%) | 607 (0.4%) | 489 (1.6%) | 1,486 (1.1%) |
| Hearing loss | 1,356 (4.4%) | 4,861 (3.4%) | 341 (1.2%) | 1,480 (1.1%) |
| Psoriasis | 1,344 (4.4%) | 4,932 (3.5%) | 367 (1.2%) | 1,435 (1.0%) |
| Sleep apnoea | 553 (1.8%) | 1,226 (0.9%) | 501 (1.7%) | 1,168 (0.8%) |
| Peptic ulcer disease | 1,421 (4.6%) | 4,253 (3.0%) | 419 (1.4%) | 1,195 (0.9%) |
| Spinal stenosis | 204 (0.7%) | 641 (0.5%) | 403 (1.3%) | 1,241 (0.9%) |
| Haematological malignancies | 257 (0.8%) | 1,067 (0.8%) | 276 (0.9%) | 1,199 (0.9%) |
| Macular degeneration | 405 (1.3%) | 1,495 (1.1%) | 298 (1.0%) | 1,161 (0.8%) |
| Dementia | 40 (0.1%) | 58 (0.0%) | 301 (1.0%) | 943 (0.7%) |
| Peripheral arterial disease | 379 (1.2%) | 1,220 (0.9%) | 262 (0.9%) | 930 (0.7%) |
| Post-viral fatigue syndrome, neurasthenia and fibromyalgia | 1,498 (4.9%) | 2,062 (1.5%) | 448 (1.5%) | 701 (0.5%) |
| Conduction disorders and other arrhythmias | 263 (0.9%) | 981 (0.7%) | 201 (0.7%) | 852 (0.6%) |
| Bronchiectasis | 184 (0.6%) | 668 (0.5%) | 246 (0.8%) | 727 (0.5%) |
| Chronic liver disease | 369 (1.2%) | 783 (0.6%) | 216 (0.7%) | 605 (0.4%) |
| Inflammatory bowel disease | 597 (1.9%) | 2,077 (1.5%) | 138 (0.5%) | 492 (0.4%) |
| Parkinson's disease | 75 (0.2%) | 240 (0.2%) | 125 (0.4%) | 470 (0.3%) |
| Epilepsy | 610 (2.0%) | 1,684 (1.2%) | 126 (0.4%) | 399 (0.3%) |
| Cardiomyopathy | 76 (0.2%) | 356 (0.3%) | 94 (0.3%) | 403 (0.3%) |
| Coeliac disease | 225 (0.7%) | 728 (0.5%) | 96 (0.3%) | 351 (0.2%) |
| Benign neoplasm of brain and other CNS | 174 (0.6%) | 524 (0.4%) | 73 (0.2%) | 358 (0.3%) |
| Meniere disease | 283 (0.9%) | 805 (0.6%) | 71 (0.2%) | 254 (0.2%) |
| Non-acute cystitis | 100 (0.3%) | 212 (0.1%) | 62 (0.2%) | 211 (0.1%) |
| Visual impairment and blindness | 111 (0.4%) | 283 (0.2%) | 65 (0.2%) | 171 (0.1%) |
| Primary pulmonary hypertension | 11 (0.0%) | 77 (0.1%) | 53 (0.2%) | 170 (0.1%) |
| Diabetes not otherwise specified | 153 (0.5%) | 534 (0.4%) | 37 (0.1%) | 133 (0.1%) |
| Asbestosis | 28 (0.1%) | 162 (0.1%) | 25 (0.1%) | 144 (0.1%) |
| Immunodeficiencies | 36 (0.1%) | 85 (0.1%) | 32 (0.1%) | 136 (0.1%) |
| HIV | 70 (0.2%) | 177 (0.1%) | 45 (0.1%) | 104 (0.1%) |
| Sarcoidosis | 102 (0.3%) | 517 (0.4%) | 20 (0.1%) | 126 (0.1%) |
| Multiple sclerosis | 203 (0.7%) | 528 (0.4%) | 37 (0.1%) | 106 (0.1%) |
| Tuberculosis | 352 (1.1%) | 1381 (1.0%) | 16 (0.1%) | 92 (0.1%) |
| Motor neuron disease | 9 (0.0%) | 28 (0.0%) | 16 (0.1%) | 91 (0.1%) |
| Type 1 diabetes | 110 (0.4%) | 438 (0.3%) | 11 (0.0%) | 46 (0.0%) |
| Myasthenia gravis | 12 (0.0%) | 68 (0.0%) | 13 (0.0%) | 42 (0.0%) |
| Addison's disease | 23 (0.1%) | 65 (0.0%) | 16 (0.1%) | 30 (0.0%) |

* Ordered by total percentage of new cases during follow-up, largest to smallest; conditions present from birth tabulated separately (Table F)

† Condition present in men only. Percentages and rates based on 68 355 men without depression, and 10 178 men with depression

CNS, central nervous system; COPD, chronic obstructive pulmonary disease; HIV, human immunodeficiency virus; LTC, long term condition;

# Table F: Number and percentage of people with a history of each long-term physical health condition at baseline, stratified by history of depression at baseline (N = 172,556). Conditions present from birth

|  | Prevalence [n (%)] | |
| --- | --- | --- |
|  | History of depression at baseline | |
| Condition* | No depression (N = 141,786) | Depression (N = 30,770) |
| Cerebral palsy | 123 (0.1%) | 29 (0.1%) |
| Thalassaemia | 115 (0.1%) | 24 (0.1%) |
| Cystic fibrosis | 65 (0.0%) | 32 (0.1%) |
| Sickle-cell anaemia | 44 (0.0%) | 9 (0.0%) |
| Down's syndrome | 21 (0.0%) | <5 (0.0%) |

* Ordered by total number of cases in the cohort, largest to smallest.

# Table G: Number and percentage of people with a history of each long-term mental health condition at baseline, stratified by history of depression at baseline (N = 172,556).

|  | Prevalence [n (%)] | |
| --- | --- | --- |
|  | History of depression at baseline | |
| Condition* | No depression (N = 141,786) | Depression (N = 30,770) |
| Anxiety disorders | 8,899 (6.3%) | 12,871 (41.8%) |
| Alcohol problems | 2,361 (1.7%) | 1,275 (4.1%) |
| Bipolar affective disorder and mania | 528 (0.4%) | 775 (2.5%) |
| Schizophrenia, schizotypal and delusional disorders | 285 (0.2%) | 406 (1.3%) |
| Other psychoactive substance misuse | 287 (0.2%) | 338 (1.1%) |
| Obsessive-compulsive disorder | 185 (0.1%) | 315 (1.0%) |
| Anorexia and bulimia nervosa | 220 (0.2%) | 262 (0.9%) |
| Post-traumatic stress disorder | 181 (0.1%) | 228 (0.7%) |
| Intellectual disability† | 166 (0.1%) | 89 (0.3%) |
| Autism and Asperger's syndrome† | 58 (0.0%) | 60 (0.2%) |

* Ordered by total number of cases in the cohort, largest to smallest.

†Condition present from birth

# Table H: Rate ratios for the association of history of depression at baseline, sociodemographic, social, lifestyle and clinical factors with physical health condition accrual during follow-up. Complete cases analysis (N = 132,394).

|  |  | **Rate ratio (95% CI)** | | |
| --- | --- | --- | --- | --- |
|  |  | **Adjusted for age and sex** | **Adjusted for sociodemographic characteristics** | **Fully adjusted** |
| History of depression at baseline |  | 1.32 (1.30, 1.34) | 1.30 (1.28, 1.32) | 1.10 (1.08, 1.12) |
| Age (rate ratio for each additional 10 years of age)* | Age | 1.59 (1.57, 1.60) | 1.60 (1.59, 1.61) | 1.43 (1.42, 1.45) |
| Sex (ref: Male) | Female | 0.80 (0.79, 0.81) | 0.81 (0.80, 0.82) | 0.83 (0.82, 0.84) |
| Socioeconomic status  Townsend deprivation decile  (ref: 1, least deprived) | 2 |  | 0.99 (0.96, 1.02) | 0.98 (0.96, 1.01) |
|  | 3 |  | 1.01 (0.98, 1.03) | 0.99 (0.96, 1.01) |
|  | 4 |  | 1.05 (1.02, 1.08) | 1.02 (1.00, 1.05) |
|  | 5 |  | 1.04 (1.02, 1.07) | 1.01 (0.98, 1.04) |
|  | 6 |  | 1.04 (1.01, 1.07) | 0.99 (0.97, 1.02) |
|  | 7 |  | 1.09 (1.06, 1.12) | 1.01 (0.98, 1.04) |
|  | 8 |  | 1.12 (1.09, 1.16) | 1.02 (0.99, 1.04) |
|  | 9 |  | 1.20 (1.16, 1.23) | 1.04 (1.01, 1.07) |
|  | 10 (most deprived) |  | 1.33 (1.29, 1.37) | 1.07 (1.04, 1.10) |
| Ethnicity  (ref: White) | Black |  | 1.18 (1.09, 1.28) | 1.05 (0.98, 1.14) |
|  | Mixed |  | 1.14 (1.03, 1.26) | 1.08 (0.98, 1.18) |
|  | South Asian |  | 1.30 (1.23, 1.37) | 1.15 (1.10, 1.22) |
|  | Other ethnic group |  | 1.10 (1.02, 1.17) | 1.05 (0.98, 1.12) |
| Country of residence at baseline  (ref: England) | Scotland |  | 0.80 (0.79, 0.82) | 0.82 (0.80, 0.83) |
|  | Wales |  | 0.99 (0.97, 1.01) | 0.95 (0.93, 0.97) |
| Morbidities at baseline* | Count |  |  | 1.06 (1.06, 1.07) |
|  | Count-squared |  |  | 1.00 (1.00, 1.00)**†** |
| Smoking  (ref: never) | Previous |  |  | 1.09 (1.08, 1.11) |
|  | Current |  |  | 1.26 (1.24, 1.29) |
| Alcohol intake frequency  (ref: daily or almost daily) | 3-4 times a week |  |  | 0.97 (0.95, 0.99) |
|  | 1-2 times a week |  |  | 1.00 (0.98, 1.02) |
|  | 1-3 times a month |  |  | 1.00 (0.98, 1.03) |
|  | Special occasions |  |  | 1.04 (1.01, 1.06) |
|  | Never |  |  | 1.08 (1.05, 1.11) |
| BMI (kg/m^2)  (ref: <25) | 25 - 29.9 |  |  | 1.10 (1.09, 1.12) |
|  | 30 - 34.9 |  |  | 1.21 (1.19, 1.23) |
|  | ≥ 35 |  |  | 1.33 (1.30, 1.36) |
| SBP (mmHg)* | log(SBP) |  |  | 1.43 (1.36, 1.50) |
|  | (log(SBP))-squared |  |  | 2.96 (2.32, 3.77) |
| Cholesterol:HDL ratio* | log(ratio) |  |  | 1.07 (1.04, 1.10) |
|  | (log(ratio))-squared |  |  | 1.26 (1.19, 1.35) |
| HbA1c (mmol/mol)  (ref: <32) | 32-34 |  |  | 0.99 (0.97, 1.01) |
|  | 34-36 |  |  | 1.02 (1.00, 1.04) |
|  | 36-38 |  |  | 1.04 (1.02, 1.06) |
|  | ≥ 38 |  |  | 1.21 (1.19, 1.24) |
| Sleeplessness/insomnia  (ref: never/rarely) | Sometimes |  |  | 1.03 (1.02, 1.05) |
|  | Usually |  |  | 1.08 (1.06, 1.10) |
| Count of stressful life events  (ref: 0) | 1 |  |  | 1.03 (1.01, 1.04) |
|  | 2 or more |  |  | 1.05 (1.02, 1.07) |
| Chronic multisite pain (ref: no) | Yes |  |  | 1.16 (1.14, 1.18) |
| Loneliness (ref: no) | Yes |  |  | 1.07 (1.05, 1.09) |
| Weight loss (ref: no) | Yes |  |  | 1.04 (1.02, 1.06) |
| Slow walking speed (ref: no) | Yes |  |  | 1.11 (1.09, 1.14) |
| Weak grip strength (ref: no) | Yes |  |  | 1.04 (1.03, 1.06) |
| Low physical activity (ref: no) | Yes |  |  | 1.07 (1.05, 1.09) |

* For each quantitative covariate, we scaled the values by subtracting their mean

**†** 0.997 (0.996, 0.997)

BMI, body mass index; HbA1c, glycated haemoglobin; HDL, high density lipoprotein; SBP, systolic blood pressure.

# References

1 Hanlon P, Nicholl BI, Jani BD, Lee D, McQueenie R, Mair FS. Frailty and pre-frailty in middle-aged and older adults and its association with multimorbidity and mortality: a prospective analysis of 493 737 UK Biobank participants. *Lancet Public Health* 2018; **3**: 323–32.
