# Supplementary material for: Depression and physical multimorbidity: A cohort study of physical health condition accrual in UK Biobank
Source: PLoS Med. 2025 Feb 13;22(2):e1004532. doi: 10.1371/journal.pmed.1004532 (PMC11825000; doi:10.1371/journal.pmed.1004532)
Supplement: Attachment — Submitted filename: fleetwood.pdf [file pmed.1004532.s003.pdf]

This paper uses data from the UK Biobank to study the association between depression and other morbidities.

I was asked for a methodological report and I interpret that to include all aspects of the design and conduct of the study.

Points of detail

Page 1 Is the word multimorbidity appropriate here and elsewhere? I interpret a study of multimorbidity as one which looks at the prevalence of multiple conditions whereas here what is being studied is the coexistence of each other condition with depression: comorbidity. I am not suggesting the authors should have studied every possible pairwise definition of multimorbidity as with 69 conditions that would be impractical.

Page 5 The problem with the definitions of prevalent and incident here is that the definition of self–reported depression could refer to a diagnosis which predated the conditions recorded at baseline which would then be incident rather that prevalent.

Page 6 If it is true that most of the missing is due to laboratory problems then one could assume missing completely at random (MCAR) and just use complete cases. This is almost the text book example of MCAR (the technician dropped the test tubes). Complete cases is usually thought to lose precision but with 100k+ participants that is hardly an issue.

Page 8 I had assumed that the analysis set was the last box but in fact it is the one above. Perhaps label the analysis set as such?

Page 11 Is this the cumulative tally of all conditions with which they have been diagnosed or is it a count of those active at any point? If you have been diagnosed with one of these conditions do you always have it? I am not a clinician but some of the conditions in Table 2 ought to be curable (anaemia? peptic ulcer disease?) or at least manageable with medication.

Page 15 I would take issue with the phrase ‘similar but somewhat weaker’. If we take the two most deprived tenths then reductions from 1.21 to 1.04 and 1.35 to 1.07 seem to me to be substantially weaker. The position with ethnicity and country is not quite so bad.

Page 18 Inserting ‘to our knowledge’ might be safer.

Supplementary Table 4 I cannot find in the text any reference to including these conditions in the models or any justification for excluding them. Perhaps I missed it.

Point of more substance

Given the very low response rate for UK Biobank ($< 6\%$) I might have expected more caution about the results. I know this is controversial but Stamatakis et al. (2021) found the results changed when they tried to weight back to the general population.

Summary

Mostly details in this extensive report of a carefully conducted study. My concerns about response rate and generalisability are doubtless old–fashioned.

Michael Dewey

References

E Stamatakis, K B Owen, L Shepherd, B Drayton, M Hamer, and A E Bauman. Is cohort representativeness *passé*? Poststratified associations of lifestyle risk factors with mortality in the UK Biobank. *Epidemiology*, 32: 179–188, 2021.
