# Supplementary material for: Depression and physical multimorbidity: A cohort study of physical health condition accrual in UK Biobank
Source: PLoS Med. 2025 Feb 13;22(2):e1004532. doi: 10.1371/journal.pmed.1004532 (PMC11825000; doi:10.1371/journal.pmed.1004532)
Supplement: Attachment — Submitted filename: Response_to_reviewers_29Nov2024.docx [file pmed.1004532.s004.docx]

# Overview

**Authors’ comment:** We would like to thank the academic editor, the editorial team and the four reviewers for taking the time to review our paper and provide helpful comments. We have responded to the reviewers’ comments below.

References to page and line numbers throughout this response refer to the revised track changes version of our manuscript.

# Reviewer #1: Michael Dewey

*This paper uses data from the UK Biobank to study the association between depression and other morbidities.*

*I was asked for a methodological report and I interpret that to include all aspects of the design and conduct of the study.*

*Points of detail*

*Page 1 Is the word multimorbidity appropriate here and elsewhere? I interpret a study of multimorbidity as one which looks at the prevalence of multiple conditions whereas here what is being studied is the coexistence of each other condition with depression: comorbidity. I am not suggesting the authors should have studied every possible pairwise definition of multimorbidity as with 69 conditions that would be impractical.*

**Authors’ response:** Our study evaluates the count of physical health conditions that participants accrued during follow-up. In most cases, where we used the word ‘multimorbidity’ we had intended to refer to ‘physical multimorbidity’, that is the co-existence of two or more long term physical health conditions. We have now clarified our definitions of ‘multimorbidity’ and ‘physical multimorbidity’ by re-writing paragraph 2 of the introduction as follows (lines 84 to 92):

Multimorbidity is defined as the coexistence of two or more long term health conditions, with physical multimorbidity specifically referring to the coexistence of two or more long term physical health conditions. Although estimates of multimorbidity prevalence differ due to large variation in the number of conditions counted [6], multimorbidity increases rapidly with age and is the norm in older people. Health and social care systems are challenged by the increasing prevalence of multimorbidity, driven by population aging, improved survival from acute conditions, and increasing incidence of chronic conditions such as type 2 diabetes [7]. However the role of depression in multimorbidity is understudied, with only half of multimorbidity studies including depression in their condition count [6]. Whilst depression has been shown to be associated with increased risk of various individual physical health conditions, less is known about its role as a risk factor for physical multimorbidity.

Where appropriate we have also updated ‘multimorbidity’ to ‘physical multimorbidity’:

- Title, line 1
- Introduction, lines 94, 102
- Discussion, lines 363, 377

*Page 5 The problem with the definitions of prevalent and incident here is that the definition of self{reported depression could refer to a diagnosis which predated the conditions recorded at baseline which would then be incident rather that prevalent.*

**Authors’ response:** Prevalence is usually defined as the number of cases/conditions at a specific point in time (in this study, the UKB baseline assessment) and incidence is usually defined as the number of new cases in a defined period (in this study, from the day after the UKB baseline assessment to the end of follow-up, which varies for each participant). Throughout the paper we have used the terms prevalent to refer to conditions diagnosed before or on each participant’s date of UKB baseline assessment and incident to refer to conditions diagnosed after this date. In response to your comment we have clarified our definition of prevalent in Methods > Outcome (page, 7, lines 175-177)

Conditions were defined as prevalent at each participant’s date of UKB baseline assessment if the condition was self-reported at the baseline assessment or the first linked record was on or before the date of the baseline assessment

It is not always possible to determine the order of diagnoses prior to baseline. Where depression or physical health conditions were identified from primary care or hospital records, we do have dates of diagnosis, however, where conditions were identified from self-report, dates of diagnosis are not always available. Furthermore, in response to Reviewer 2’s first point we have clarified that our exposure is ‘history of depression at baseline’, and updated the text throughout the manuscript, which may also help to clarify how we have defined our exposure and outcome.

*Page 6 If it is true that most of the missing is due to laboratory problems then one could assume missing completely at random (MCAR) and just use complete cases. This is almost the text book example of MCAR (the technician dropped the test tubes). Complete cases is usually thought to lose precision but with 100k+ participants that is hardly an issue.*

**Authors’ response:** Although cholesterol:HDL ratio and HbA1c had the highest proportions of missing data, there was missing data in a further 15 covariates which are not laboratory measurements. Hence the most robust approach was to conduct a multiple imputation.

In Table 1 we originally did not report missing data for variables where data was missing for less than one percent in each group. Such variables were indicated with an asterisk. We initially took this decision in order to make the baseline characteristics table as short as possible. To make the extent of missing data clearer we have now updated Table 1 to include these rows (pages 12 to 14).

*Page 8 I had assumed that the analysis set was the last box but in fact it is the one above. Perhaps label the analysis set as such?*

**Authors’ response:** Thank you for the suggestion. We have now labelled the second last box in Figure 1 as ‘Primary analysis: multiple imputation’

*Page 11 Is this the cumulative tally of all conditions with which they have been diagnosed or is it a count of those active at any point? If you have been diagnosed with one of these conditions do you always have it? I am not a clinician but some of the conditions in Table 2 ought to be curable (anaemia? peptic ulcer disease?) or at least manageable with medication.*

**Authors’ response:** Figure 2 illustrates the cumulative tally of all conditions with which participants have been diagnosed. We have now expanded our methods section to include a description of this figure (page 8, lines 216 to 219)

To provide a simple visualisation of the accrual of long-term physical health conditions, we plotted the cumulative mean number conditions per participant from baseline to end of follow-up by age, sex and history of depression at baseline. The cumulative mean at each time point was based on participants followed up until at least that time point.

We have also clarified the title and the vertical axis of this figure by exchanging ‘mean’ for ‘cumulative mean’. In our analyses, we do not allow for recovery or remission of conditions. We selected long-term conditions but naturally there will be some cases where participants recover or go into remission, however it is very challenging to identify recovery or remission from electronic health records. In response to this point we have also updated the Methods > Outcome section as follows (page 7, lines 181-183):

Since we focused on long-term health conditions, our analyses did not allow for recovery or remission from conditions. Furthermore, such information is not well recorded in the electronic health records.

*Page 15 I would take issue with the phrase ‘similar but somewhat weaker’. If we take the two most deprived tenths then reductions from 1.21 to 1.04 and 1.35 to 1.07 seem to me to be substantially weaker. The position with ethnicity and country is not quite so bad.*

**Authors’ response:** In response to Reviewer 4, point 11, as our aim was to determine the association between depression and the accrual of long-term physical health conditions we have now removed the paragraphs (page 19, lines 330-343) about the relationship between the other covariates and the accrual of conditions.

*Page 18 Inserting ‘to our knowledge’ might be safer.*

**Authors’ response:** We have updated the first sentence of the final paragraph of the discussion section to “In the most comprehensive study to date on this topic, to our knowledge, we identified a higher rate of accrual for comorbid physical health problems in people with a history of depression compared to those without.” (page 22, line 451)

*Supplementary Table 4 I cannot find in the text any reference to including these conditions in the models or any justification for excluding them. Perhaps I missed it.*

**Authors’ response:** Supplementary Table 4 (Table G in Supplementary Information S2 Text, in the revised version) describes the number and percentage of people with each long-term mental health condition at baseline (excluding depression). It is referenced in the Results section, page 9, line 268. Our count of conditions accrued during follow-up does not include mental health conditions, however mental health conditions were included in our count of conditions present at baseline. We have made the following clarifications in our revised text:

- In the original version of the supplement, a single table listed both the long-term physical and mental health conditions that we measured in our study. We have now separated this into two separate tables in the supplement: Table B includes long-term physical health conditions, and Table C includes long-term mental health conditions.
- In Methods > Outcome, page 7, line 180-181 we have clarified that our outcome is long-term physical health conditions.
- In Methods > Covariates, pages 7 to 8, lines 202 to 207, we have more carefully described our count of conditions present at baseline: “For each participant, we calculated a total (prevalent) condition count at baseline. This count included the 69 physical health conditions used in the outcome and additionally ten non-depression mental health conditions (Table C in S2 Text). We ascertained these mental health conditions from baseline assessment, primary care and hospital data using the same approach that we used to ascertain the physical health conditions.”

*Point of more substance*

*Given the very low response rate for UK Biobank (< 6%) I might have expected more caution about the results. I know this is controversial but Stamatakis et al. (2021) found the results changed when they tried to weight back to the general population.*

**Authors’ response:**

We have now updated the section in the discussion that mentioned the limitations of using the UK Biobank cohort as follows (pages 20 to 21, lines 386 to 394):

Only 5.5% of people invited to join the UK Biobank participated in the baseline assessments, with participants less likely on average to live in deprived areas, and more likely to have better health compared with the general population [32]. Baseline condition counts and rates of accrual of new conditions are therefore likely to underestimate values in the general population. Furthermore, our estimates of the association between history of depression at baseline and physical health condition accrual may not be generalisable to the wider population [33,34]; however, it is reassuring that other multimorbidity associations estimated from the UK Biobank cohort were generally similar to associations estimated from a nationally representative sample [35].

*Summary*

*Mostly details in this extensive report of a carefully conducted study. My concerns about response rate and generalisability are doubtless old{fashioned.*

*References*

*E Stamatakis, K B Owen, L Shepherd, B Drayton, M Hamer, and A E Bauman. Is cohort representativeness pass´e? Poststratified associations of*

*lifestyle risk factors with mortality in the UK Biobank. Epidemiology, 32:179{188, 2021.*

# Reviewer #2

*Authors present a study examining the association between history of depression and the accumulation of physical diseases using data from UKB. Using a large study population with a wide age range at baseline (40-71 years), they report a faster rate of disease accumulation in people with a history of depression. These associations are preserved after accounting for ethnicity, area-level deprivation, and country of residence. Subsequent adjustment for clinical, behavioural, and functional indicators, as well as prevalent diseases, reduces the effect of depression history, although it remains. The paper is well-written and the analysis is sound. I have some concerns about conceptualizing depression, lack of age-group stratification given the wide age-span of the sample, and overall innovativeness of the study, given the many studies examining depression-multimorbidity connection out there.*

*1.      Authors are not consistent in their terminology/conceptualization of depression. In some sections, they speak about prevalent depression (eg., people with/without depression), whereas in most other places, they write "history of depression". In my reading, their operationalization of depression is more consistent with the notion of "depression history", at least when it comes to the self-report item. For the electronic records this is less clear, and more detail is required from authors here. Does depression assessment from the records only refer to the last eight years, or is it possible to gauge the history of depression from these data? Depending on the answer here, the conceptualization of depression diagnosis will vary. Consequently, if one source of depression diagnosis used here refers to history (self-repot), while the other to the last eight years, then combining them into a single measure may not be suitable. Please provide more detail about conceptualizing depression from the different sources and consider sensitivity analysis with alternative operationalizations of depression (using just one source of diagnosis?) to limit misclassification and ensure consistency between operational definition and its conceptualization.*

**Authors’ response:** You’re correct that our exposure is history of depression. We sometimes wrote ‘depression’ rather than ‘history of depression’ for brevity. We have now clarified the text:

- Abstract > Conclusions (page 2, line 43). “without depression” replaced with “without a history of depression”
- Introduction (page 5, line 116). “depression” replaced with “history of depression”
- Results (page 9, lines 258, 259, 260, 262, 264, 265; page 10, lines 270, 272, 273; page 14, lines 291, 293, 294; page 17, lines 314, 315, 317, 318, 320. “depression” replaced with “a history of depression”
- Discussion (page 19, line 347; page 22, line 429): “depression” replaced with “history of depression”
- Table 1 caption (page 12, line 283): “depression status” replaced with “history of depression at baseline”
- Table 1 (page 12): Additional header row added: “History of depression at baseline”
- Figure 2 caption (page 15, line 301): “depression” replaced with “history of depression”
- Figure 2: Updated legend with a title “History of depression at baseline”
- Supplement S2 Text
- Table E (previously Table 2 in the main text) caption (page 10): “depression” replaced with “history of depression”
- Table E: Additional header row added: “History of depression at baseline”
  - Table F and Table G
    - Updated caption with ‘history of depression’
    - Additional header row added: “History of depression at baseline”
  - Table H: Updated the caption and row label for depression to read ‘history of depression at baseline’

We have also provided more information about how we defined history of depression from the electronic health records in the Methods > Depression section (page 7, lines 185 to 193). The text now says:

“Our exposure is history of depression at baseline: defined as a diagnosis of depression at any time before on the participant’s UKB baseline assessment date. In the United Kingdom depression is most often diagnosed in primary care, and primary care records should capture a participant’s entire medical history, however in order to capture as many depression cases as possible, we defined history of depression based on multiple sources. We identified diagnoses of depression from linked primary care or hospital records, or self-reported depression in response to the baseline assessment question “Has a doctor ever told you that you have had any other serious medical conditions or disabilities?”.”

*2.      What is the overlap between the self-report of depression and the diagnosis from the electronic records?*

**Authors’ response:** We have now added additional information about the source of the depression diagnoses to the Results section (page 9, lines 254 to 257):

History of depression at baseline was predominately identified from primary care records; 90.7% of cases had a primary care diagnosis on or before baseline, 4.2% had a hospital diagnosis on or before baseline, and 31.5% of cases self-reported a previous diagnosis of depression.

To illustrate the overlap we have also added a proportional Venn diagram to the supplementary material (S2 Text, Figure A).

*3.      Did the authors consider episodic nature of depression in their operationalization? Is it handled in any way, particularly for data from electronic records? At the very least, this should be discussed in the limitations.*

**Authors’ response:** We hope that the changes to our Methods section in response to your first comment have clarified how we defined depression. Our exposure is history of depression at baseline, rather than active depression. Whilst understanding the relationship between active depression and the accrual of long-term physical health conditions is also of interest, the available data does not allow us to reliably capture the remission and relapse of depression throughout the follow-up period.

We have updated the limitations (page, 21, lines 407-409) to read “Finally, we were unable to examine the role of depression remission and relapse and depression severity because such information was not reliably captured by the data.”

*4.      Authors are working with a very wide age range (40-71 years at baseline). We know that both multimorbidity and depression are age-sensitive syndromes, increasing in prevalence with age (multimorbidity), or exhibiting a more chronic course and poor prognosis, with an altered clinical presentation (depression). Indeed, some have argued that old-age depression may be conceptually distinct from middle-aged depression, with an outsized role played by somatic and cognitive symptoms (as opposed to affective/low mood ones). Given such age-dependency in both exposure and outcome, I think it is imperative to not lump all ages together, but rather conduct stratified analysis and discuss age-specific findings at length.*

**Authors’ response:** We agree that additional analyses stratified by age could have been a worthwhile addition to our paper, however, unfortunately we no longer have access to the participant level data. UK Biobank data is provided for use within a defined time period and unfortunately our access ended earlier this year. In response to this point, and your following point, we have added the following sentence to our discussion (page 22, lines 438 to 442):

We estimated the association in a broad cohort including both men and women, ranging in age from 40 to 71 years at baseline. However, it would also be valuable to explore this association in analyses stratified by key sociodemographic characteristics such as age and sex.

We would also like to draw your attention to Figure 2, which illustrates the cumulative mean number of long-term physical health conditions at baseline and during follow-up by history of depression at baseline, age (in three strata) and sex. This figure illustrates a relatively consistent gap between people with and without depression, despite baseline differences in age and sex.

*5.      To a large extent, this also applies to sex. In fact, authors themselves mention sex differences in the introduction, therefore it would only be natural to revisit them later on in the paper*.

**Authors’ response:** In line with our response to point 4 above, we agree that additional analyses stratified by sex could have been a worthwhile additional to our paper, however, unfortunately we no longer have access to the participant level data.

*6.      For the operationalization of chronic conditions, I have several points. 1) Did the authors consider dropping hypertension from disease count and re-calculating accrual rates without it? Some in MM literature have argued that hypertension represents a risk factor rather than an overt disease, and given its high prevalence, the inclusion of hypertension could be misleading. 2) I was unclear about the mental conditions other than depression... Were they considered as part of the total count in the accrual analysis? If they were, authors should do a sensitivity analysis without them, considering the comorbidity of mental disorders, particularly depression and anxiety (especially in older adults). 3) please consider providing baseline prevalences of diseases in the Supplementary Table 1.*

**Authors’ response:** 1) We included hypertension in line with recommendations from the Delphi consensus study conducted by Ho et al. (2022) (reference 18 in the main text), although we recognise that hypertension is sometimes considered a risk factor rather than a condition. We have added the following sentence to our discussion (page 20, lines 379 to 382):

However, we acknowledge that there is ongoing debate about what conditions should be included in multimorbidity measures, and whether some conditions, such as hypertension, should instead be treated as risk factors.

2) Mental health conditions other than depression were included in the count of prevalent morbidities at baseline, but not in the count of conditions accrued during follow-up. We have now made the following clarifications in our revised text:

- In the original version of the supplement, a single table listed both the long-term physical and mental health conditions that we measured in our study. We have now separated this into two separate tables in the supplement: Table B includes long-term physical health conditions, and Table C includes long-term mental health conditions.
- In Methods > Outcome, page 7, line 180 we have clarified that our outcome is long-term physical health conditions.
- In Methods > Covariates, pages 7 to 8, lines 202 to 207, we have more carefully described our count of conditions present at baseline: “For each participant, we calculated a total (prevalent) condition count at baseline. This count included the 69 physical health conditions used in the outcome and additionally ten non-depression mental health conditions (Table C in S2 Text). We ascertained these mental health conditions from baseline assessment, primary care and hospital data using the same approach that we used to ascertain the physical health conditions.”

3) Baseline prevalences of the long-term physical health conditions are now provided in Table E in S2 Text, with the exception of conditions present from birth, where baseline prevalences are given in S2 Text Table F. Baseline prevalences of mental health conditions are also given in the supplement S2 Text Table G.

*7.      The inclusion of stressful life events, loneliness, pain, and sleep could be problematic, considering their possible conceptual overlap with depression (indeed sleeping difficultly is one of depression symptoms). This is further complicated by the fact they are assessed concurrently with depression. I suggest their inclusion is reconsidered.*

**Authors’ response:**

As noted above, we unfortunately no longer have access to the participant level data so we cannot change the final model. We agree that there is possibly some conceptual overlap between the above covariates and depression. We have added the following sentence to our limitations paragraph (page 21, lines 396 to 400):

Whilst our partially adjusted models included information on covariates that likely preceded both our exposure and outcome and thus acted as confounding factors, some covariates included in our final model may lie on the causal pathway or have a conceptual overlap with depression. Thus, our final model may have underestimated the strength of the association between depression and physical condition accrual.

*8.      Indeed, the analysis in which factors on the causal pathway between depression and disease count are considered as a way of explaining away depression's effect is not well justified in the introduction. As I read, the aim seems to be about predictive effects of depression on multimorbidity change, whereby depression and MM are kept temporally and conceptually distinct to strengthen inferences. This analysis, blurs this conceptual separation, and requires better justification.*

**Authors’ response:** At the start of our statistical analysis section we have now clarified our aim (page 8, lines 214 to 215):

Our aim was to describe to what extent the rate of accrual of long-term physical health conditions differs between people with and without depression.

As noted above in response to point 7, we now also highlight the limitations of the final model. The partially adjusted model may better represent the association between depression and physical disease accrual, if covariates included in the fully adjusted model are considered to be on the causal pathway.

*9.      How much of the drop in the effect of depression is attributable to controlling for prevalent conditions?*

**Authors’ response:** We did not compare the model adjusted for sociodemographic characteristics to a model adjusted only for sociodemographic characteristics and the count of prevalent conditions at baseline. As stated above, we no longer have access to the participant level data, so we cannot conduct this additional analysis. In terms of more carefully identifying the causal pathway between depression and the accrual of long-term physical health conditions, we have now added the following to our discussion section (page 22, lines 442 to 446):

Further research should also more carefully explore the causal relationship between depression and the subsequent accrual of long-term physical health conditions in order to identify potential interventions, including whether effective management of depression and/or more assertive management of risk factors for physical disease in people with depression leads to lower rates of physical disease accrual.

*10.     I have doubts about the usefulness of the results in Table 2 according to prevalent/incident individual diseases. To me, this goes back to lacking age-group perspective. Ignoring huge age differences runs the risk of misrepresenting these cross-tabs. Also, in the limitations, authors write that examining the relationship between depression and specific physical conditions was outside the scope of this study. Still, they devoted a lengthy table 2 to individual diseases. I think the paper would benefit more from an explicit age/sex perspective, considering the size of authors' dataset.*

**Authors’ response:** We have now moved this table to the S2 Text Table E.

*11.     In the face of lacking age/sex/disease type/depression severity/course perspective, I am left wondering about the place of this study in the field where depression-multimorbidity associations have been extensively researched before (and synthesized in previous reviews, including scoping and systematic ones). Undoubtedly, authors address some previous gaps (primarily study size and depth of multimorbidity assessment), although they are confronted with others such as depression conceptualization issues and non-representativeness of the study population. And given the lack of an "angle" that helps triangulate these findings a specific and well-defined at-risk subpopulation, I am left wondering exactly what new this study brings to the table (or, to rephrase it, whether should it be published in a high-impact journal like PLOS Medicine).*

**Authors’ response:** We hope that our response to your 1^st^ point has clarified how we conceptualised depression. Reviewers 1 and 4 also commented on the representativeness of the UKB cohort, and we have addressed this by expanding the section in our discussion that mentioned its limitations as follows (pages 20 to 21, lines 386 to 394):

Only 5.5% of people invited to join the UK Biobank participated in the baseline assessments, with participants less likely on average to live in deprived areas, and more likely to have better health compared with the general population [32]. Baseline condition counts and rates of accrual of new conditions are therefore likely to underestimate values in the general population. Furthermore, our estimates of the association between history of depression at baseline and physical health condition accrual may not be generalisable to the wider population [33,34]; however, it is reassuring that other multimorbidity associations estimated from the UK Biobank cohort were generally similar to associations estimated from a nationally representative sample [35].

Whilst we agree that there is already considerable research in the area of depression and multimorbidity, much of this research has been cross-sectional rather than longitudinal, as highlighted in our Introduction (lines 102 to 103). Whilst several studies have investigated the association between baseline depression and the accrual of subsequent physical health conditions, to the best of our knowledge, we include more conditions than any previous study. In many of the previous studies, participants were followed up through intermittent surveys where there is a high risk of loss to follow-up; whereas our use of electronic health records avoids this limitation, and particularly the use of primary care data allows us to identify diseases that are usually treated in primary care rather than secondary care. For these reasons we hope that our study will be of interest to readers of PLOS Medicine.

# Reviewer #3

*This paper tackles a highly relevant topic, investigating the relationship between depression and the accumulation of physical health conditions (multimorbidity) using the UK Biobank cohort. The study is well-structured, leverages a robust dataset, and contributes valuable insights to the growing body of literature on mental health and its intersection with chronic disease. The paper demonstrates methodological rigor and offers important implications for public health practice and clinical care. However, there are several areas where the manuscript can be improved to enhance clarity, rigor, and interpretability of results.*

*One question relates to the choice of covariates:*

*The authors include a lot of covariates in their analysis, and the justification for selecting many of these is not clear. Were they chosen because of theoretical importance?*

**Authors’ response:** The fully adjusted model adjusts for a broad set of covariates. We have now highlighted the limitations of this model in our discussion (page 21, lines 396 to 400):

Whilst our partially adjusted models included information on covariates that likely preceded both our exposure and outcome and thus acted as confounding factors, some covariates included in our final model may lie on the causal pathway or have a conceptual overlap with depression. Thus, our final model may have underestimated the strength of the association between depression and physical condition accrual.

We would like to highlight that our partially adjusted models only adjust for a small set of core sociodemographic characteristics. The partially adjusted models may better represent the association between depression and physical disease accrual, if covariates included in the fully adjusted model are considered to be on the causal pathway.

# Reviewer #4

*This study addresses an important research topic on the impact of depression on the subsequent accrual of physical health conditions. The strengths of the work include its large cohort size (172,556) participants and the comprehensive inclusion of conditions in the definition of multimorbidity - which set this paper aside from many of the existing studies in the literature at present.  Whilst at face value the analyses is well conducted and presented, in particular, a high quality imputation approach as been implemented - I do have several major concerns regarding the overall analyses and modelling approach which are critical to the overall reliability and quality of the results.*

*1. Introduction - paragraph on multimorbidity and depression studies being cross-sectional references many of the key papers, however it is incomplete and could be more balanced therefore. e.g. There are studies which look at disease accrual over time/in sequence (e.g. Hayward et al. Lancet eBioMedicine, 2023 Volume 96, 104792 - disease trajectories) which shows data on the time sequenced accrual of mental health related hospital admissions following cardiovascular diseases and its association with increased risk of death. A more comprehensive review of the literature to highlight the novel contribution of this work therefore is recommended.*

**Authors’ response:** In our introduction, our aim, in line with PLoS Med guidelines, was to provide a ‘brief review of the key literature’. Since the main focus of this paper is depression and the subsequent accrual of physical health conditions, we briefly mentioned a couple of the key cross-sectional studies. We then focused on studies that reported on depression and the subsequent accrual of physical conditions. Hayward et al. (2023) examines trajectories of major disease diagnoses post myocardial infarction, whilst we examine the accumulation of diseases post depression. Whilst the baseline disease is different, the methodological approach is relevant, and we have added a reference to Hayward et al. in our discussion (page 22, lines 434 to 436):

Investigating the ordering of subsequent long-term physical health conditions [39] would also be worthwhile, although it is often difficult to establish the order of conditions from electronic health records, especially where multiple diagnoses are recorded on the same day.

*2. Whilst UK Biobank is a fantastic resource - the disadvantages of its use for multimorbidity research must be highlighted up front. The authors use strong language in the introduction to suggest they "robustly" identify physical conditions, and whilst I appreciate they have gone over and above existing studies in their attempts to be much more inclusive and comprehensive in accrual of conditions than others - the level of selection bias and healthy user bias inherent in UK Biobank does limit the robustness of this assessment. Further linked primary care data is available for fewer than 50% of the cohort. Further data on the level of linkage to other data sources do not feature strongly (or at all?) - and these all affect the level of confidence in ascertainment and the equality of ascertainment of both depression and the physical conditions for individuals in the study. Moreover over half the cohort are excluded given the lack of primary care linkage - I realise this is a necessary choice given the data but this does introduce further queries over the representativeness of these data and its conclusions which need to be fully acknowledged as limitations. The language in the intro and discussion need to be appropriately moderated to reflect this also.*

**Authors’ response:** In response to this point, and reviewer 1’s final point we have expanded our text on the limitations of using the UK Biobank cohort as follows (page 20, lines 386 to 394):

Only 5.5% of people invited to join the UK Biobank participated in the baseline assessments, with participants less likely on average to live in deprived areas, and more likely to have better health compared with the general population [32]. Baseline condition counts and rates of accrual of new conditions are therefore likely to underestimate values in the general population. Furthermore, our estimates of the association between history of depression at baseline and physical health condition accrual may not be generalisable to the wider population [33,34]; however it is reassuring that other multimorbidity associations estimated from the UK Biobank cohort were generally similar to associations estimated from a nationally representative sample [35].

In response to this point, we have also removed the word ‘robustly’ from our introduction (page 5, line 118). We have now clarified in our Methods section (page 5, lines 137 to 138) that “Linked primary care data was available from Scotland, Wales and practices in England that used either the TPP or Vision practice management systems.” Hence, where no linked primary care data is available for a participant, this is usually because they were registered with a practice that used a different management system. We have extensively revised our linked electronic health records section (page 6, lines 145 to 160). This now specifies that “all UKB participants are linked to hospital, cancer registry and death records” (lines 145 to 146).

*Statistical analyses*

*3. High quality imputation approach has been used (which is rare - and therefore commended)! - the approach follows good practice guidelines for MICE such as including the outcome variable and all covariates and their formulations, as well as a comparison with complete cases. Please state whether any additional auxiliary variables were included in the imputation models or not, and include a detailed description of missing data for all variables (e.g. by an extra column in Table 1). Missing data are reported for some but not all variables in the table - e.g. it is hard to believe there were no missing data at all for BMI and alcohol intake - yet missing data for these are not included in Table 1.*

**Authors’ response:** Thank you for your positive feedback on our multiple imputation approach.

No additional auxiliary variables were included in the imputation models. Given the large number of sociodemographic, clinical, social and lifestyle variables already included in the model we felt that it was unnecessary to include additional auxiliary variables. We have now clarified that no auxiliary variables were included in the text (page 9, lines 244 to 246): “They did not include any auxiliary variables because the covariates already covered a broad set of sociodemographic, clinical, social and lifestyle factors”.

In the original version of Table 1 (as noted in its footnote) we did not report missing data for variables where data was missing for less than one percent in each group. Such variables were indicated with an asterisk. We originally took this decision in order to make the baseline characteristics table as short as possible. We have now updated Table 1 to include these rows (pages 12 to 14).

*4. Table 2 presents details of those with and without depression according to the conditions at baseline and those accrued during the follow up period. It is not clear however what the timing of those baseline conditions in relation to depression is? They can be before or after the depression as far as I can tell - which makes interpretation of this table difficult. Could the authors elaborate on this? How does this impact on your results?*

**Authors’ response:** In this study we use the UK Biobank baseline assessment as the start point. The second and third columns of this table (Table E in S2 Text in the revised version) summarise the conditions present at this start point. Amongst participants with depression and physical health conditions at baseline, we do not identify the ordering of these conditions, because this is not always possible; where depression or physical health conditions were identified from primary care or hospital records we do have dates of diagnosis, however, where conditions were identified from self-report, dates of diagnosis are not always available.

We have now clarified the baseline by updating our descriptions of prevalent conditions:

- Methods > Outcomes (page 7, lines 175-177): Conditions were defined as prevalent at each participant’s date of UKB baseline assessment if the condition was self-reported at the baseline assessment or the first linked record was on or before the date of the baseline assessment
- Methods > Covariates (page 7, lines 202-203): For each participant, we calculated a total (prevalent) condition count at their UKB baseline assessment date

We also updated the caption and column headers of Table E in S2 Text by replacing ‘baseline’ with ‘UKB baseline assessment’.

Columns 2 and 3 of Table E in S2 Text should be interpreted as a cross-sectional report of prevalence of each long-term physical health condition in those with and without a history of depression at baseline.

*5. "Baseline depression" has been identified as any occurrence of depression recorded during an 8 year medical history - however this is likely to lead to a heterogenous population with differential impact on outcomes for e.g. individuals who had one short episode of depression related to an acute event vs. those with longer term/persistent depression or mental health conditions over the length of follow up. Has this been accounted for as these two scenarios are likely to influence the impact of depression physical health conditions very differently.*

**Authors’ response:** In response to this question, and a similar question from Reviewer 2, we have clarified how depression was defined in the Methods > Depression section (page 7, lines 185 to 193). We have not accounted for the severity of depression, or remission and relapse of depression. This was outside the scope of this study, because such information was not reliably captured by the available data. We have expanded our text in the limitations sections as follows (page 21, lines 407 to 409):

**“**Finally, we were unable to examine the role of depression remission and relapse and depression severity because such information was not reliably captured by the data.**”**

*6. Observational studies of this type should at minimum use some form of matching between cohorts to minimise additional sources of bias. I would expect to see either a propensity score analyses, or other form of matching (e.g. risk-set matching approach) minimise bias between the groups  (esp. given concerns about ascertainment highlighted below).*

**Authors’ response:** We hope that our responses to your comments 2, 4, and 5 have clarified our study design. Given our study design, covariate adjustment is an appropriate method to estimate the association between history of depression at baseline and the accrual of long term physical health conditions during follow-up.

*7. Given that ascertainment of depression and physical health conditions will vary hugely between primary, secondary care and the UK biobank baseline data it is important to understand how many cases included in the study were linked with the various datasets and over what time periods these linkages were present. Those with fully linked data may have an increase in physical health conditions recorded and a higher chance of a record for depression vs those who have fewer linked datasets and it would be important to know the degree with which this varies for individuals in the cohort. How does this impact on your results, and has this been accounted for in any way?*

**Authors’ response:** In response to this point, and point 2 above we have extensively revised our linked electronic health records section (page 6, lines 145 to 160). This now specifies that “all UKB participants are linked to hospital, cancer registry and death records” (lines 145-146). We have also added the dates of data availability to the supplement, Table A.

*8. The use of centring and quadratics in the modelling is a little outdated and requires either further justification plus clear model diagnostics to be presented (e.g. why was age divided by 10 specifically? How much improvement in model fit were you looking for in the quadratics? centring is usually done to aid interpretation, but as these are just included as confounders and should not to be directly assessed (See point later about "table 2 fallacy" - there seems little point). This approach overall is a little inefficient and requires a number of steps and judgements to be made in the process. Accounting for non-linearity in variables is better done via the use of restricted cubic splines which estimates all transformations simultaneously (instead of adding quadratic terms for all predictors and then determining which ones should be kept). This approach would also lead to appropriate confidence intervals of your estimates - which are not influenced by scale of the data and therefore do not require centring.*

**Authors’ response:** Based on our experience of large observational studies where multiple imputation is required we take a practical approach to statistical modelling, using the simplest appropriate method. Whilst we agree that modelling continuous variables using restricted cubic splines allows for more flexibility than adding quadratic terms, in this study, we found that a linear relationship was adequate to describe the relationship between age and rate of accrual for long-term physical health conditions, and quadratic relationships were sufficient to describe the relationships between each of the count of morbidities at baseline, log systolic blood pressure and log cholesterol:HDL ratio, and the rate of accrual of conditions. We divided age by 10 to aid our interpretation of the rate ratio for age (this will, of course, make no difference to the estimation of the rate ratio for depression), and used centring to avoid multicollinearity between each continuous outcome and its square. It is worth noting that, with the exception of age, these continuous covariates are only included in the fully adjusted model.

*9. The modelling approach does not appear to take different follow up periods for individuals and any censoring into account (as far as I could discern from the paper). Lack of accounting for this will cause major biases in your model which should not be published unless addressed (e.g. at minimum through inclusion of an appropriate offset term in the Poisson model, but ideally through use of a time to event modelling framework). In the current analyses it is far more likely to identify someone in the "depression" group if they have more follow up time (i.e. there is more opportunity to identify their existing diagnoses) and therefore any comparisons between a depressed and non-depressed group is likely to be inherently biased. In addition to that - the outcome (number of conditions accrued) is heavily influenced by length of follow up time and censoring - which therefore must be addressed in the modelling directly.*

**Authors’ response:** Thank you for identifying this issue. We included an offset term in the quasi-Poisson models, however this detail was accidentally omitted from the statistical analysis section. We have now added it in (page 8, lines 222 to 223): “We accounted for each participant’s follow-up time by including the log follow-up time as an offset term in the models.”

*10. The inclusion of a good number of confounders is commended - however - further thought needs to be provided as to why those variables are indeed included. Were other data available and discounted, are all these variables definitely confounders and not mediators? The use of a DAG in this instance of many variables collected for a dataset such as these is highly recommended, which would also identify any further potentially important confounders which were not available to be listed in the discussion.*

**Authors’ response:** The fully adjusted model includes some covariates which may potentially lie on the causal pathway. We have added the following sentence to our limitations paragraph (page 21, lines 396 to 400):

Whilst our partially adjusted models included information on covariates that likely preceded both our exposure and outcome and thus acted as confounding factors, some covariates included in our final model may lie on the causal pathway or have a conceptual overlap with depression. Thus, our final model may have underestimated the strength of the association between depression and physical condition accrual.

We would like to highlight that our partially adjusted models only adjust for a small set of core sociodemographic characteristics. The partially adjusted models may better represent the association between depression and physical disease accrual, if covariates included in the fully adjusted model are considered to be on the causal pathway.

*11. The authors present their modelling data in such a way that falls foul of the so called "Table 2 Fallacy" - which is when estimates of confounders are presented and interpreted directly as exposures in their own right whilst adjusting for all other confounders. The only relevant estimates to be interpreted and presented for this particular model is the RRs for depression vs no depression. It's fine to include a model with several "layers" of confounders, although STROBE/RECORD guidance recommends the reporting of unadjusted RRs in addition to these also. The RRs for all the confounders do not have / should not be interpreted independently in any way and therefore should not be presented in the table or described as they are in the results section. The only act as confounders in this case.  If the authors are additionally interested in the effects of age on morbidity accrual - then a new model should be generated with age as the main exposure and a bespoke set of confounders relevant to the age-outcome relationship should be identified, and this should also be added to the original aims of the presented work.*

**Authors’ response:** We have now removed the paragraphs that discussed the associations between the other covariates and the accrual of long-term physical health conditions from our results section, page 19, lines 330-343.

*12. Figure 2 shows a plot of mean number of long term conditions - this is a simplistic view of these data which may be biased by not accounting for differential follow up time for individuals contributing to these data, and confidence intervals should be displayed for these curves also. The use of formal time to event analyses accounting directly for censoring and length of follow up time is advised here (e.g. calculating cumulative incidence functions) and displayed with confidence intervals. It is also good practice to list the numbers at risk in time intervals (e.g. for each year of follow up) for each of these plots so that the accuracy of the longer term data (which is a stated strength of this paper). There will be fewer individuals with longer term follow up data - and the drop off rate of this should be clearly presented.*

**Authors’ response:** We hope that our manuscript will appeal to a wide audience, including clinicians and policymakers without specialist statistical knowledge. We included Figure 2 specifically to provide a simple view of the data, and the footnote below this figure highlights that the mean at each time point is based only on individuals followed up until at least that time point. The results of the quasi-Poisson models provide a formal analysis of the data, account for censoring and length of follow-up time, and include confidence intervals. Since this plot includes sub-plots by sex and age group and separate means for participants with and without a history of depression at baseline, we have not included confidence intervals or numbers at risk in order not to complicate the plot. In the methods section, we now clarify the purpose of this plot (page 8, lines 216 to 219):

To provide a simple visualisation of the accrual of long-term physical health conditions, we plotted the cumulative mean number conditions per participant from baseline to end of follow-up by age, sex and history of depression at baseline. The cumulative mean at each time point was based on participants followed up until at least that time point.

*13. Aside from the table 2 fallacy as already discussed - the strength of association cannot be compared in this way with respect to size of estimate; therefore this statement and others similar throughout the manuscript should be removed : " The association between depression and the accrual of physical conditions was of a similar magnitude to associations between other characteristics and condition accrual, although weaker than associations with age, smoking, and BMI in the fully adjusted model".*

**Authors’ response:** We have now removed the above sentence from our manuscript (Discussion, page 19, lines 350-353) and additionally removed “and a higher rate of accrual of physical conditions subsequently” (Discussion, page 22, line 426).
