# Supplementary material for: Depression and physical multimorbidity: A cohort study of physical health condition accrual in UK Biobank
Source: PLoS Med. 2025 Feb 13;22(2):e1004532. doi: 10.1371/journal.pmed.1004532 (PMC11825000; doi:10.1371/journal.pmed.1004532)
Supplement: Attachment — Submitted filename: Response to requests from editors_9Jan2025.docx [file pmed.1004532.s005.docx]

# Overview

**Authors’ comment:** We would like to thank the editorial team and the reviewers for taking the time to reread our paper. We have responded to the requests from the editors below.

References to page and line numbers throughout this response refer to the revised track changes version of our manuscript.

# Requests from Editors

1. *Abstract, line 34-35 and line 36: Please refrain from expressing RRs as fold (or times)-increase; suggest modifying to “…participants with depression accrued physical morbidities at a faster rate than those without depression (RR 1.32…)…”*

**Authors response:** We have updated the text in the abstract (lines 34 to 38) as follows:

After adjustment for age and sex, participants with depression accrued physical morbidities at a faster rate than those without depression (RR 1.32, 95% confidence interval [CI] [1.31, 1.34]). After adjustment for all sociodemographic characteristics, the rate of condition accrual remained higher in those with versus without depression (RR 1.30, 95% CI [1.28, 1.32]).

We have also updated similar wording in the results section (lines 287 to 295) as follows:

Based on all participants in our cohort, in the unadjusted model, people with a history of depression accrued new long-term physical health conditions at a faster rate than people without a history of depression (rate ratio [RR] 1.25, 95% confidence interval [CI] [1.23, 1.27]). After adjustment for age and sex, the rate of condition accrual remained higher in people with versus without a history of depression (RR 1.32, 95% CI [1.31, 1.34]) (table 2). Additional adjustment for sociodemographic factors only slightly attenuated the effect estimate (RR 1.30, 95% CI [1.28, 1.32]). In the fully adjusted model, which included potential mediators, a history of depression was associated with a marginally increased rate of morbidity accrual (RR 1.10, 95% CI [1.09, 1.12]).

*2. Abstract. Please include a sentence describing the main limitation(s) of the study at the end of the Methods and findings section.*

**Authors response:** We have added the following sentence to the end of the Methods and findings section:

The main limitation of this study is healthy volunteer selection bias, which may limit generalisability of findings to the wider population.

*3. For references to URLs (eg, ref 7, 21), please add the date accessed in brackets.*

**Authors response:** We have addressed this by verifying that the URLs in the references are up to date and adding today’s date to the references (references 1, 7, 21).

*4. Please add a URL to the sponsor’s website to your funding statement.*

**Authors response:** We have added the URLs for the MRC and NIHR to the funding statement (lines 410 to 411).

*5. Please add a URL for UK Biobank to your Data availability statement.*

**Authors response:** We have added the URL for UK Biobank (line 420).
